# Atomistic Simulation Study of Nanoparticle Effect on Nano-Cutting Mechanisms of Single-Crystalline Materials

**DOI:** 10.3390/mi11030265

**Published:** 2020-03-04

**Authors:** Pengyue Zhao, Qi Zhang, Yongbo Guo, Huan Liu, Zongquan Deng

**Affiliations:** 1 School of Mechatronics Engineering, Harbin Institute of Technology, No. 92, Xidazhi Street, Nangang District, Harbin 150001, China; liuhuanxues@163.com (H.L.); zhaopengyue129@126.com (Z.D.); 2 Qian Xuesen Laboratory of Space Technology, No. 104, Youyi Road, Haidian District, Beijing 100094, China

**Keywords:** nanoparticle, nano-cutting, plastic deformation, surface quality, molecular dynamics

## Abstract

Nanoparticle (NP), as a kind of hard-to-machine component in nanofabrication processes, dramatically affects the machined surface quality in nano-cutting. However, the surface/subsurface generation and the plastic deformation mechanisms of the workpiece still remain elusive. Here, the nano-cutting of a single-crystalline copper workpiece with a single spherical embedded nanoparticle is explored using molecular dynamics (MD) simulations. Four kinds of surface/subsurface cases of nanoparticle configuration are revealed, including being removed from the workpiece surface, moving as a part of the cutting tool, being pressed into the workpiece surface, and not interacting with the cutting tool, corresponding to four kinds of relative depth ranges between the center of the nanoparticle and the cutting tool. Significantly different plastic deformation mechanisms and machined surface qualities of the machined workpiece are also observed, suggesting that the machined surface quality could be improved by adjusting the cutting depth, which results in a change of the relative depth. In addition, the nanoparticle also significantly affects the processing forces in nano-cutting, especially when the cutting tool strongly interacts with the nanoparticle edge.

## 1. Introduction

Nano-cutting, as one of the most efficient micro/nanoscale manufacturing techniques, is widely applied in micro-electro-mechanical system (MEMS) and nano-electromechanical system (NEMS) to produce components with micro/nanoscale surface roughness or feature size [1,2,3]. In general, the crystal orientation of the workpiece is carefully selected and optimized to adjust the direction of cutting chips accumulation and ensure the surface quality of the workpiece [4]. However, at the nanoscale, the cutting process could be affected by very small defects, such as the microstructural components [5], pores/voids [6], second phase substances [7], and nanoparticles [8], leading to poor machined surface quality. Note that such small defects, especially the substances/nanoparticles, would significantly affect the machined surface generation [7], microstructural evolution, and processing force in nanofabrication [8], resulting from the strong interaction between the substances/particles and the tool. Although high purity materials almost without defects, like amorphous nickel-phosphorous [9], exist, materials with defects still play a significant role in the nanofabrication because of the specific requirements of the properties of the workpiece, such as strength, stiffness, and thermal stability [10]. Hence, studies on the nanofabrication mechanisms of difficult-to-machine materials with defects, like the nanoparticle (NP), are necessary to improve the machined surface quality of the workpiece.

NP, as a kind of special hard-to-machine component in the workpiece, plays a great role in the plastic deformation mechanisms of the workpiece in nanofabrication, which draws a lot of attention [11,12]. Due to the fact that the NP is generally used to enhance the mechanical properties of some special materials, for example, metal matrix composites [13,14], it can exist in the surface or subsurface of the raw materials in nanofabrication. Ge et al. [15] conducted ultra-precision turning tests to investigate the surface/subsurface motion of the NP in the metal matrix composites, deeming that the distance from the NP to material free surface determines the NP be pulled out or pressed into the machined surface. The research by Ding et al. [16] suggests that NPs play an important role in the void generation of the machined workpiece. Xu et al. [17] found that the NP could be removed as a whole entity or be fractured into several pieces because of the strong interaction between the NP and the cutting tool, and the fractured NP pieces could enlarge the void regions on the machined surface. The NPs have an important influence on the mechanical behaviors of the workpiece in nanofabrication, but the research on the nano-cutting mechanism of the workpiece with NPs is still lacking.

Compared with the high cost and time consuming experimental method, the molecular simulation method [18,19] can give an in-depth atomic analysis and is widely used to investigate the plastic deformation mechanism in nanomachining, including the microstructural evolution [20], processing force [21], internal stress [22], atomic motion [23], surface generation [24], equivalent stress [25], elastic modulus [26], and friction behaviors [27]. Therefore, it is necessary to study the surface/subsurface motion of the NP in nano-cutting.

In this work, we investigate the surface/subsurface motion of the NPs in a single-crystalline copper workpiece using large-scale molecular dynamics (MD) simulations. By adjusting the relative depths between the cutting tool and the center of the NPs, four different cases are observed for the surface/subsurface motion of the NPs in nano-cutting, resulting in significantly different subsurface defects, internal stress distribution, and processing force of the machined workpiece. The results are potentially essential to reveal the relative machined surface quality of the workpiece with NPs during the nano-cutting process.

## 2. Simulation Methods

**Figure 1:**
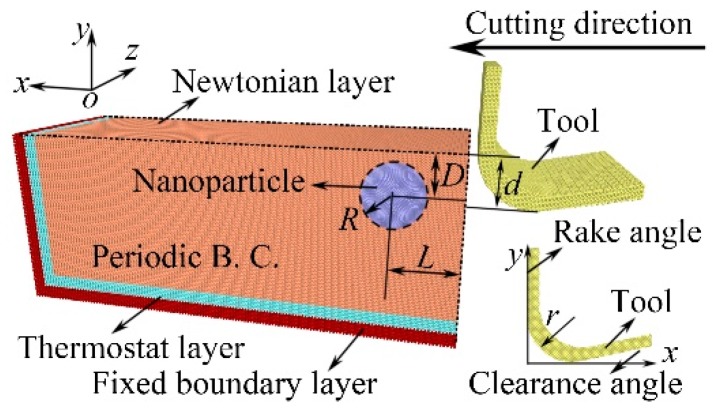
Nano-cutting molecular dynamics (MD) simulation model with a nanoparticle.

The nano-cutting processes of the workpiece with an NP at different depths beneath the surface are performed by MD simulations. Due to the fact that the material is generally removed in a limited range smaller than the grain size [28], the material of the workpiece is set to single-crystalline copper with a lattice constant of 3.615 Å. The nano-cutting model consisting of a single-crystalline copper workpiece, a rigid diamond tool [29], and a diamond NP [30] is shown in Figure 1. For simulation convenience, the NPs are simplified as spherical shape. The radius *R* of the NP is 4.0 nm. The distance *L* from the right side of the workpiece to the center of the NPs is 10.0 nm. The depth *D* is the distance between the center of the NPs and the surface of the workpiece. The cutting depth *d* is 5.0 nm. The relative depth Δ*D* is defined as Δ*D* = *d* − *D*, and the Δ*D*s from −5.0 nm to 5.0 nm (every 1.0 nm) are adopted in this work.

The cutting tool has a rake angle of 0° and a clearance angle of 12° [31]. The edge radius *r* of the cutting tool is 4 nm. The workpiece with a dimension of 60.0 nm × 30.0 nm × 20.0 nm is categorized into three layers, containing the Newtonian layer where the atoms follow Newton’s law, thermostat layer where the atoms are kept at a constant temperature, and fixed boundary layer where the atoms are fixed to prevent the unexpected atomic motion, respectively. The z-direction of the system is prescribed as a periodic boundary condition (Periodic B. C.) to eliminate the simulation size effect [32]. The cutting tool is applied 1 nm from the right side of the workpiece. Before nano-cutting, the workpiece is adjusted to energy minimization using the conjugate gradient method. Thereafter, the workpiece is relaxed at 293 K and 1 atmosphere using a Nose-Hoover thermostat in the NPT ensemble for 100 ps. Finally, the NVE ensemble is applied to the equilibrated workpiece during the nano-cutting [33]. The time step is 1 fs. The nano-cutting direction is {110} <001>. The nano-cutting speed is 50 m/s, i.e. 0.5 Å/ps. The embedded atom method (EAM) potential is used to describe the Cu–Cu interactions [34,35], and the Tersoff potential [36] is used to describe the C–C interactions. The Morse potential is adopted to describe the Cu–C interactions [37,38], as shown in Equation (1).

(1) $$E(r)=D_{0}\left[\exp(-2\alpha (r-r_{0}))-2\exp(-\alpha (r-r_{0}))\right]\;r<r_{c},$$

where *E* is the potential energy; *D*_0_ is the binding energy (0.1 eV); *α* is the elastic modulus (1.70 Å^−1^); *r*_0_ is the atomic spacing (2.20 Å); and *r*_c_ is the cutoff (5.00 Å) [39,40]. All MD simulations are executed by the open-source code large-scale atomic/molecular massively parallel simulator (LAMMPS) [41]. The defects in the workpiece are identified by the dislocation extraction algorithm (DXA) [42]. The twin boundary (TB) is denoted by a single hcp layer, and the stacking fault (SF) (containing intrinsic stacking fault (ISF) and extrinsic stacking fault (ESF)) is denoted by two adjacent hcp coordinated layers [43]. The atomic displacement vector is obtained based on the program open visualization tool (OVITO) [44].

There are two parts of the validation of the MD model in this work, including the rationality of the size of the nano-cutting MD model and the correctness of simulation parameters in the MD simulation. For the size of the nano-cutting MD model, the the size of the workpiece at the cutting direction (x-direction) is large enough for the sufficient interaction between the workpiece and the NPs, and the size of the workpiece at the z-direction is eliminated by using periodic boundary condition, which is validated in both Xu [17] and Zhang [29] works; hence, the size of the nano-cutting MD model is also reasonable. For the parameters in the MD simulation, in both Xiao [45] and Zhang [46,47] works, the EAM potential for Cu-Cu system and Morse potential for Cu-C system are used in the nano-cutting MD simulation, and the MD simulation results are validated through the experimental results in details. In addition, the NPT ensemble for Cu-Cu system relaxation and the NPT ensemble for the nano-cutting MD simulation in this work are also consistent with such works. Hence, the potentials and ensembles used in this work are reasonable.

## 3. Results and Discussions

### 3.1. Mechanical Response

**Figure 2:**
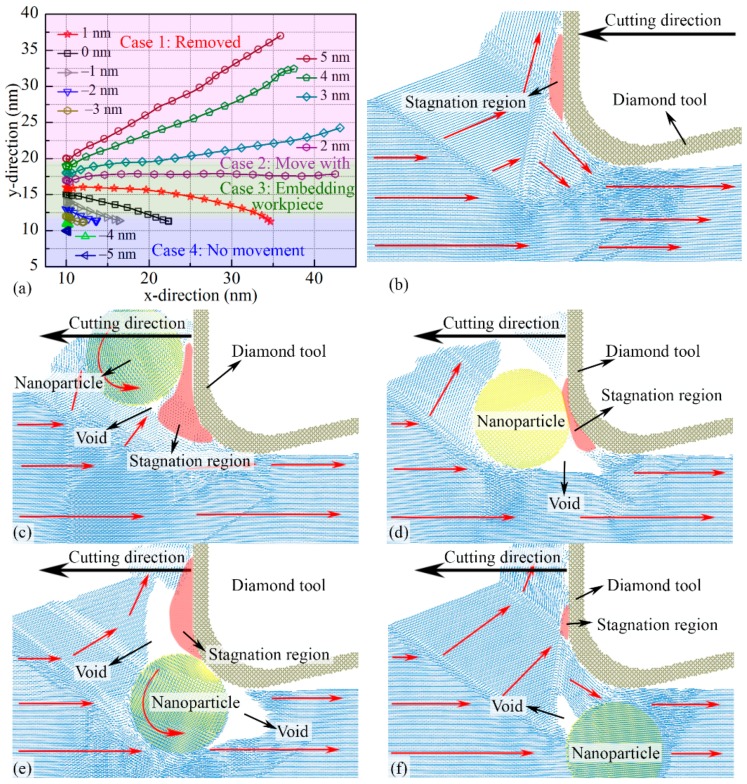
Position evolution of the center of the nanoparticle (NP) for different Δ*D*s in the x-y plane during the nano-cutting process (**a**). The atomic motion vector in the workpiece with no NPs (**b**), with NP for Δ*D* = 4 nm (**c**), 2 nm (**d**), 0 nm (**e**), and −5 nm (**f**) at cutting distance of 15 nm.

In nano-cutting, the NP moves and rotates at the cutting direction as a result of the interaction between the cutting tool and the NP. The trajectories of the NPs in the x-y plane for different Δ*D*s are shown in Figure 2a. Because the periodic B. C. is adopted at z-direction, the NP motion at z-direction is not discussed. The trajectories are divided into four cases, and the corresponding atomic motion vector of the workpiece for each case is shown in Figure 2b–f, respectively. The red arrows point to the atomic motion direction in atomic motion figures. The hcp structure layers like TB and ISF are colored in red. In nano-cutting the single-crystalline copper without NPs, as shown in Figure 2b, a TB in front of the cutting tool propagates to the workpiece free surface along the {111} plane and divides the surface into two parts, which can be treated as the primary deformation plane [48]; another TB is beneath the clearance face of the cutting tool. The TBs are divided into several parts by the Shockley partial dislocations, and some of the Shockley partial dislocations meet and form the stair-rod dislocations beneath the cutting tool [49]. A few ISFs form on the {111} plane surrounding the cutting tool. The angle from such ISFs to the {110} <001> cutting direction is 36.3°. A stagnation region forms in front of the cutting tool, where the atomic displacement vectors tend to be zero. The stagnation region usually works as a part of the cutting tool and interacts with the workpiece surface in nano-cutting. Note that the range and shape of the stagnation region are always changing. The stagnation region divides the workpiece surface atoms into two parts: the atoms above the stagnation region flow along with the cutting tool, forming the nano-cutting chips; the atoms below the stagnation region are pressed into the workpiece subsurface by the clearance face of the cutting tool, forming the machined surface. 

From Figure 2a, the surface/subsurface motion of the NP in nano-cutting can be distinguished into four cases based on the value of Δ*D*: Case 1 (NP with Δ*D* > 2 nm), the strong interaction between the NP and the nano-cutting chips in front of the cutting tool leads to the NP rolling upwards and moving out from the workpiece surface. The cutting tool has not directly contacted with the NP, but the stagnation region between the NP and the cutting tool pushes the NP rolling upwards, as shown in Figure 2c; Case 2 (NP with Δ*D* = 2 nm), instead of moving away from the workpiece free surface, the distance between the NP and the cutting tool at y-direction is constant. It means that the NP works as a part of the cutting tool to press and divide the workpiece surface atoms. Hence, the atoms in front of the NP are cut into two parts: some of the atoms move upwards along the edge of NP, forming the nano-cutting chips, and the other atoms are pressed into the workpiece subsurface, forming the machined surface. More ISFs are observed beneath the NP than those beneath the NP with Δ*D* > 2 nm. Due to the NP moving along the cutting direction without rolling, a long pore region forms behind NP, as shown in Figure 2d; Case 3 (NP with −4 nm < Δ*D* < 2 nm), as the cutting tool contacting to the NP, the NP is subjected to high compressive pressure at y-direction from the cutting tool, leading to the NP embedding into the workpiece. The NP works as a new rake face of the cutting tool, participating in the nano-cutting process, pushing the atoms in front of the NP upwards. These atoms flow away from the workpiece surface and form the nano-cutting chips. Finally, a large pore forms behind the NP, which seriously affects the machined surface roughness, as shown in Figure 2e; and Case 4 (NP with Δ*D* ≤ −4 nm), the TB is not affected by the NP, and no other TBs form surrounding the NP. The number of Shockley partial dislocations, stair-rod dislocations, TBs, and ISFs is also similar to that in the workpiece with no NPs. Although a small pore forms in front of the NP, the NP stays at the initial position since it does not contact the cutting tool, as shown in Figure 2f. We selected four typical Δ*D*s that contain 4 nm, 2 nm, 0 nm, and −5 nm in the above four cases to discuss the NP effect on the nano-cutting process, respectively. 

### 3.2. Plastic Deformation Mechanism

**Figure 3:**
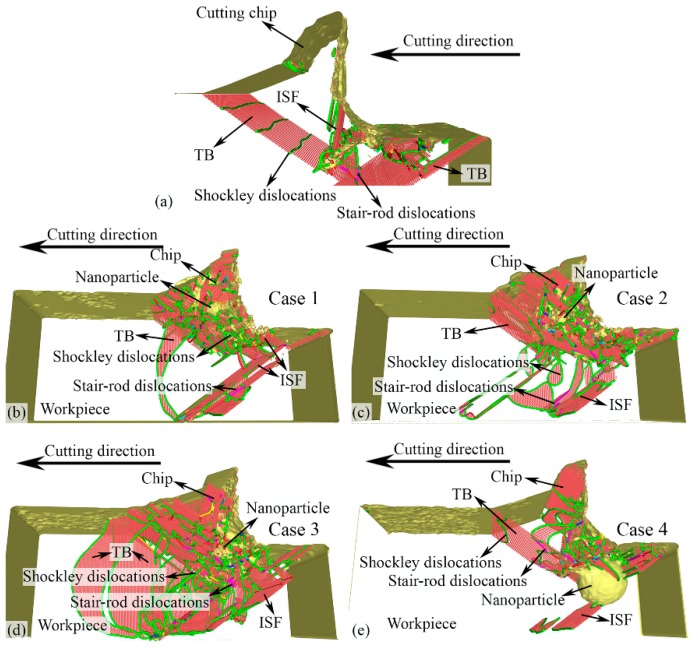
Snapshots of the defects in the subsurface of the workpiece with no NPs (**a**), with NP for Δ*D* = 4 nm (**b**), 2 nm (**c**), 0 nm (**d**), and −5 nm (**e**) at cutting distance of 15 nm.

The defects at the cutting distance of 15 nm in the surface/subsurface of the workpiece with different Δ*D*s corresponding to the four cases are shown in Figure 3, where the fcc atoms are hidden and the hcp and other types of crystal structure atoms are colored in red and gray, respectively. The perfect dislocations, Shockley partial dislocations, and stair-rod dislocations, are colored in blue, green, and purple, respectively. 

In nano-cutting for NP with Δ*D* = 4 nm, the TB expansion is not affected by the NP, until it reaches the NP edge. Thus, only a part of the TB expands to the workpiece free surface, as shown in Figure 3b. Compared to the results from Figure 3a, more TBs and ISFs form surrounding the NP. In addition, surrounding the NP, a number of Shockley partial dislocations nucleate and propagate, which locate at the edge of the TBs and ISFs. In addition, the Stair-rod dislocations surrounding the NP region form by the meeting Shockley partial dislocations [50,51]. In the nano-cutting for NP with Δ*D* = 2 nm, the TB expansion is also affected by the NP, and with the increase of cutting distance, more TBs form surrounding the NP, as shown in Figure 3c, which is similar to the results in the nano-cutting for NP with Δ*D* = 4 nm. The TBs are divided into several parts by the Shockley partial dislocations. A few stair-rod dislocations are observed surrounding the NP in this high internal stress region. In the nano-cutting for NP with Δ*D* = 0 nm, the NP is pressed into the workpiece subsurface by the cutting tool, resulting in a large number of TBs, SFs, and various types of dislocations forming in the plastic deformation region, as shown in Figure 3d. In the nano-cutting for NP with Δ*D* = −5 nm, due to the fact that the position of the NP is lower than the cutting depth, the cutting tool does not drive the motion of the NP, as shown in Figure 3e. From the above results, the NPs with −4 nm < Δ*D* ≤ 2 nm lead to poor machined subsurface quality and should be avoided in the actual nanofabrication.

**Figure 4:**
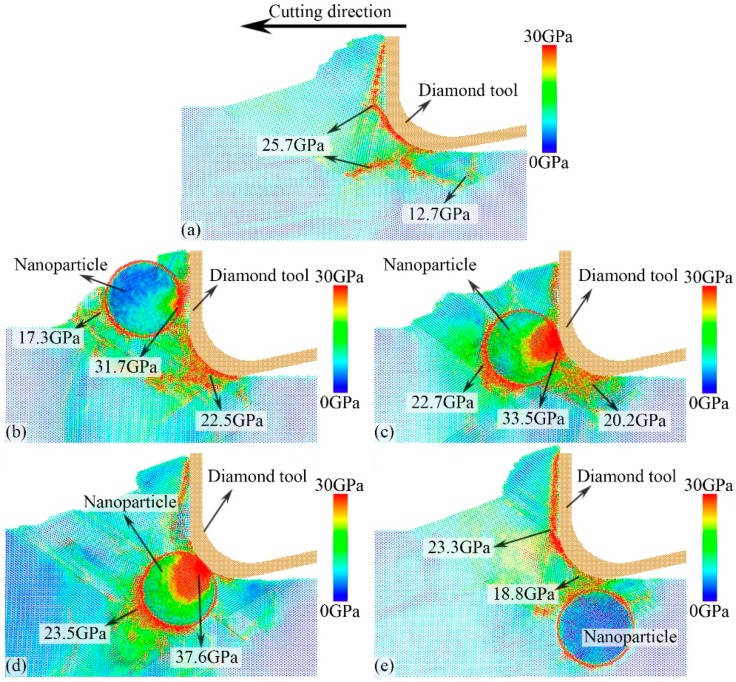
Snapshots of the von Mises stress distribution in the subsurface of the workpiece with no NPs (**a**), with NP for Δ*D* = 4 nm (**b**), 2 nm (**c**), 0 nm (**d**), and −5 nm (**e**) at cutting distance of 15 nm.

Figure 4 shows the distribution of the von Mises stresses for different cases at a cutting-distance of 15 nm. In the nano-cutting of the workpiece with no NPs, the strong interaction between the cutting tool and the free surface of the workpiece leads to high von Mises stresses in front of the cutting tool. The von Mises stresses at the edge of the stagnation region are higher than those both inside the stagnation region and in the plastic deformation region of the workpiece. This is because the atoms in the stagnation region are relatively stationary and can be treated as a new rake face of the cutting tool interacting with the workpiece in nano-cutting [52]. Hence, the von Mises stresses at the edge of the stagnation region are the highest, and the maximum von Mises stress is 25.7 GPa. The von Mises stress in the machined surface region behind the clearance face of the cutting tool is less than 3 GPa, as shown in Figure 4a. This is because of the sufficient release of the internal stress of the machined surface, although there is still a small amount of residual stress [53,54].

In the nano-cutting of NP with Δ*D* = 4 nm, the cutting tool pushes the workpiece surface atoms forward, leading to high von Mises stress between the NP and the cutting tool. The von Mises stress transfers into the workpiece subsurface on the {111} plane, which is similar to the expansion of the ISFs. It means that the high internal stress in the plastic deformation region could transfer with the defect expanding, such as the TBs and ISFs. Then, the von Mises stress reaches the NP surface, leading to the NP rolling and being removed from the workpiece surface. Only the NP edge that contacts the cutting tool exhibits high von Mises stress, while the von Mises stress in the other side of the NP trends to zero, as shown in Figure 4b. The maximum von Mises stress is 31.7 GPa. In the nano-cutting for NP with Δ*D* = 2 nm, the strong interaction between the cutting tool edge and NP leads to the right side of the NP exhibiting high von Mises stress. Because the cutting tool pushes the NP moving but without rolling in the cutting direction, the left part of the NP would push the workpiece surface atoms, leading to high von Mises stresses in the plastic deformation region. The direction of the von Mises stress gradient on both sides of the NP is parallel to the cutting direction. Hence, the NP would not move upward and be removed from the workpiece, or move downward and be pressed into the workpiece, but it moves along the cutting direction with the cutting tool, as shown in Figure 4c. The maximum von Mises stress is 33.5 GPa, which exists in the contact region between the cutting tool and the NP. In the nano-cutting for NP with Δ*D* = 0 nm, similar to the results of the nano-cutting for NP with Δ*D* = 2 nm, high von Mises stress forms on both sides of the NP. However, the direction of the von Mises stress gradient is obliquely downward, leading to the NP continuously being pressed into the workpiece subsurface. During the nano-cutting process, a large number of atoms are extruded out of the workpiece free surface, forming more nano-cutting chips (compared with the other three cases). Hence, the internal stress in the plastic deformation region is the highest in the above four cases, and the maximum von Mises stress is 37.6 GPa. In the nano-cutting for NP with Δ*D* = −5 nm, the von Mises stress transmission during the nano-cutting process is similar to the results in the workpiece with no NPs. When the cutting tool moves near the top of the NP, the internal stress transmission would be blocked by the NP and such blocked and absorbed internal stresses have not changed the internal stress inside the NP, as shown in Figure 4e. 

### 3.3. Machined Surface Quality

**Figure 5:**
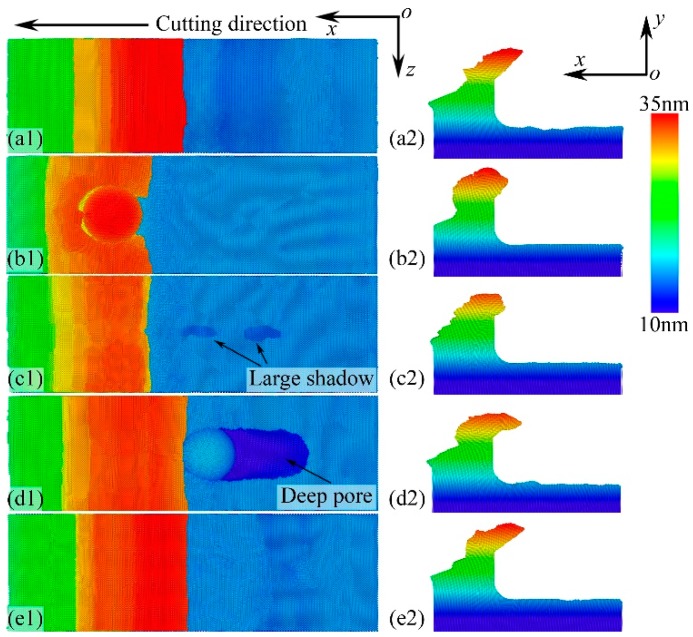
Machined surface quality of the workpiece with no NPs (**a**), with NP for Δ*D* = 4 nm (**b**), 2 nm (**c**), 0 nm (**d**), and −5 nm (**e**) under the workpiece free surface.

Figure 5 shows the machined surface qualities resulting from the motion of the NPs for the four different cases. The machined surface of the workpiece with no NPs is smooth and has no pores, as shown in Figure 5a. In the nano-cutting for NP with Δ*D* = 4 nm, where the NP moves out of the surface, the machined surface is similar to that of the workpiece with no NPs. The NP interacts with the cutting tool and rapidly rolls along with the nano-cutting chips away from the workpiece surface. The removed NP can be observed in the nano-cutting chips, as shown in Figure 5b. However, in the nano-cutting for NP with Δ*D* = 2 nm, where the NP moves as a part of the cutting tool, a large shadow is seen in the workpiece machined surface. The NP rolls at the cutting direction with the cutting tool, leaving a long pore on the machined surface. Although the material tends to fill the pore, a small pore still exists on the machined surface. Note that, due to the fact that the NP always moves with the cutting tool, many pores are formed on the machined surface. In the nano-cutting for NP with Δ*D* = 0 nm, where the NP embeds into the workpiece, a large and deep pore forms in the machined surface. The NP is pushed into the workpiece subsurface in nano-cutting. Meanwhile, a large amount of material is removed from the workpiece surface. In the nano-cutting for NP with Δ*D* = −5 nm, the NP is not influenced by the cutting tool and stays at its initial position, as shown in Figure 5e. 

### 3.4. Processing Force

**Figure 6:**
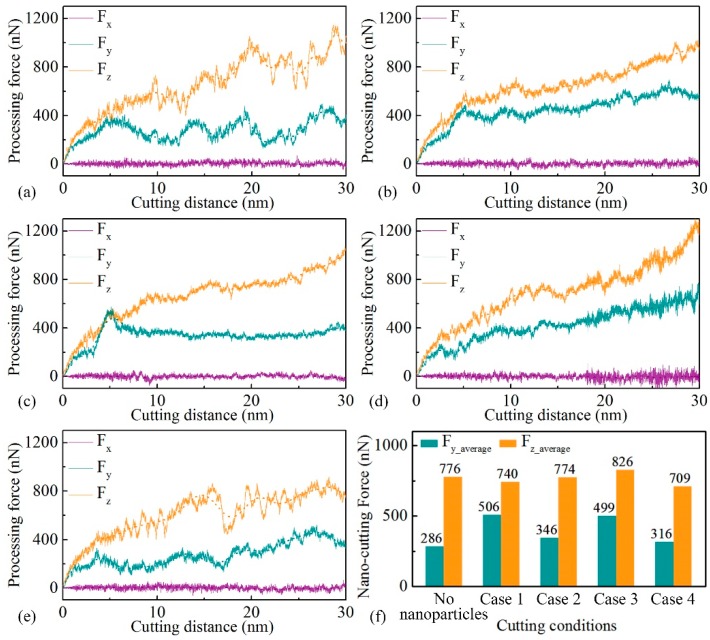
Evolution of the nano-cutting force of the workpiece with no NPs (**a**), with NP for Δ*D* = 4 nm (**b**), 2 nm (**c**), 0 nm (**d**), and −5 nm (**e**), and the mean nano-cutting force at x- and y-directions (**f**).

Figure 6 shows the evolution of processing force components, i.e., the tangential force/cutting force (x-direction), normal force (y-direction), and lateral force (z-direction). Both the cutting force and normal force increase with the cutting distance (<5 nm), and then they reach a stable cutting stage (>10 nm), as shown in Figure 6a–e. The three components of the processing force fluctuate because of the defect evolution in the workpiece. The mean value of the processing force in x- and y-directions at the stable stage is calculated, as shown in Figure 6f. The lateral force has a mean value of zero during the nano-cutting process due to the balanced and opposing forces from the two sides of the groove. The workpiece atoms above the TB in front of the cutting tool is pile-up, which would be pressed upwards by the cutting tool, forming the nano-cutting chips; meanwhile, the atoms below the TB are pressed into the workpiece subsurface, forming the machined surface. Hence, the cutting force is the highest, followed by the normal force, and the lateral force is lowest.

Compared with the cutting force in the workpiece with no NPs in Figure 6a, the interaction between the NP and the TB in the nano-cutting for NP with Δ*D* = 4 nm leads to a higher normal force (approximately 2 times), as shown in Figure 6b. In the nano-cutting for NP with Δ*D* = 2 nm, at the beginning of the nano-cutting, the normal force quickly increases due to the strong interaction between the NP and the cutting tool. It reaches the peak value at the cutting distance of 5 nm, and then it decreases to a relatively stable value. A similar cutting force is attained compared with the NP with Δ*D* = 4 nm. In the nano-cutting for NP with Δ*D* = 0 nm, due to the fact that the NP is pressed into the workpiece subsurface, both the cutting force and normal force rapidly increase with the cutting distance, as shown in Figure 6d. The processing force curve for the NP with Δ*D* = −5 nm is similar to that of the workpiece with no NPs. The mean value is also similar to that in the workpiece with no NPs. This is because the processing force is not strongly influenced by the NP, and the NP stays at the initial position during the nano-cutting process. Hence, to obtain a better processing force in nano-cutting, the NP with Δ*D* = −5 nm should be selected by choosing a lower cutting depth than the position of the NPs.

## 4. Conclusions

The selection of appropriate cutting depth is important and meaningful for the nano-cutting process to obtain better-machined surface quality and less residual stress of the workpiece with several embedded nanoparticles. The nano-cutting processes of the single-crystalline copper with a nanoparticle at different depths relative to the workpiece surface are systematically investigated using molecular dynamics simulations. At a fixed nano-cutting depth, four kinds of nano-cutting mechanisms are observed. Only when the center of the nanoparticle is higher than the edge center of the cutting tool can the nanoparticle be removed from the workpiece surface. In other cases, the nanoparticles are left inside the workpiece, moving as a part of the cutting tool, being embedded deeper by the cutting tool, or not interacting with the cutting tool. A large and deep pore remains in the workpiece surface after the nanoparticle is pressed down into the workpiece by the cutting tool, which should be avoided in the nano-cutting process for better surface quality. Very high internal stress generates in the region close to the contact area when the cutting tool collides with the nanoparticle. The spread of the high stress along the moving direction at the nanoparticle–workpiece interface causes the generation and propagation of the defects so that more defects are found in the workpiece in which the nanoparticles are left and have interacted with the cutting tool. 

Hence, in nano-cutting, to remove the nanoparticle from the workpiece surface, the nanoparticles with a relative depth Δ*D* greater than 2 nm should be selected by choosing a higher cutting depth than the position of the nanoparticles; to obtain a better processing forces in nano-cutting, the nanoparticle with a relative depth Δ*D* less than −4 nm should be selected by choosing a lower cutting depth than the position of the nanoparticles; and to obtain better machined surface quality, the nanoparticle with a relative depth Δ*D* greater than 2 nm or the nanoparticle with a relative depth Δ*D* less than −4 nm should be selected by choosing a lower/higher cutting depth than the position of the nanoparticles.
